# Pregnancy impacts allergy‐related differences in the response to a type‐1 stimulus, staphylococcal enterotoxin A

**DOI:** 10.1002/clt2.70007

**Published:** 2024-10-26

**Authors:** Claudia Arasa, Niamh Hyland, Caroline Nilsson, Eva Sverremark‐Ekström

**Affiliations:** ^1^ Department of Molecular Biosciences The Wenner‐Gren Institute Stockholm University Stockholm Sweden; ^2^ Department of Clinical Science and Education, Södersjukhuset Karolinska Institutet Stockholm Sweden; ^3^ Sachs' Children and Youth Hospital Stockholm Sweden

To the Editor,

*Staphylococcus (S*.*) aureus* is an intermittent or permanent skin colonizer in 90% of patients with airway diseases, and staphylococcal enterotoxin‐IgE serum levels have been linked to both allergy and severe asthma.^1^, ^2^ During pregnancy, immune adaptation is required to ensure fetal growth,^3^ and type 2 responses are enhanced. These changes potentially worsen allergic conditions and increase the susceptibility to certain infections.^4^

Here we investigate the immune response to Staphylococcal enterotoxin A (SEA), a strong inducer of type 1 responses, in individuals with Th2‐skewing,^5^ using peripheral blood mononuclear cells (PBMC) from allergic and non‐allergic, pregnant and non‐pregnant women^6^ (Figure 1A). Staphylococcal enterotoxins cause polyclonal T cell activation crosslinking the MHC‐II on antigen‐presenting cells (APCs) to the T‐cell receptor (TCR) on T‐cells (Figure 1B), leading to a strong proinflammatory response, potentially increasing IgE‐production or disrupting the maternal‐fetal tolerance.

**FIGURE 1:**
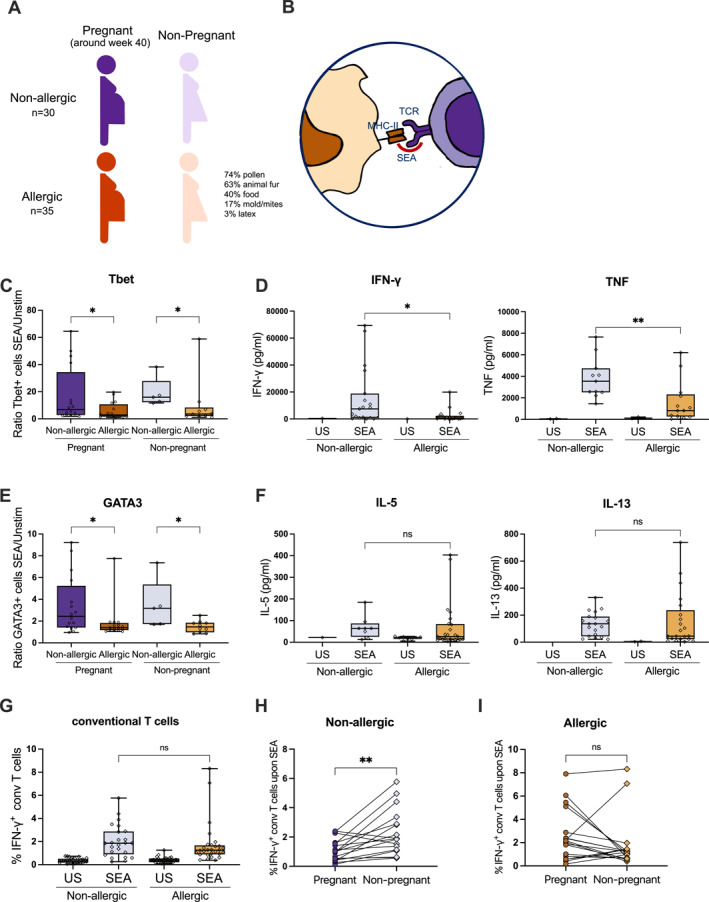
T cell responses to SEA. (A) Graphical representation of the cohort and the donors used. (B) Superantigen presentation to T‐cells by APCs. PBMCs were cultured and stimulated with SEA for 48h: (C) Ratio of CD4+ T‐cells expressing Tbet normalized to the unstimulated. (D) Secreted levels of IFN‐γ and TNF. (E) Ratio of CD4+ T‐cells expressing GATA3 normalized to the unstimulated. (F) Secreted levels of IL‐5 and IL‐13. (G) Proportion of IFN‐γ‐producing conventional T cells. Longitudinal assessment of the IFN‐γ production upon SEA during (○) and out of pregnancy (◊) in non‐allergic (H) and allergic (I) women. (*n* = 5–28).

Allergic individuals exhibited reduced Tbet expression (Figure 1C), associated with Th1 response, and lower type 1 cytokine production (IFN‐γ and TNF; Figure 1D). These differences were not observed during pregnancy (Figure S1A). GATA3 expression, linked to Th2 responses, was lower in allergic individuals regardless of their pregnancy status (Figure 1E), but there was no difference in type 2 cytokine secretion (IL‐5 and IL‐13; Figure S1B and Figure 1F). Type 3‐ and regulatory T cell markers (RORγt or FoxP3 expression and IL‐17 and IL‐10 secretion, respectively) did not differ in any of the groups (Figure S1C,D). Analyzing IFN‐γ and TNF production in conventional T cells outside of pregnancy showed comparable IFN‐γ levels between allergic and non‐allergic individuals (Figure 1G). During pregnancy, IFN‐γ production was significantly reduced in non‐allergic individuals (Figure 1H) but not in allergic (Figure 1I). TNF production was lower in allergic individuals, but it increased during pregnancy (Figure S2A).

We have previously shown that the response to SEA by unconventional lymphocytes is delayed, and that their activation strongly contributes to the elicited cytokine storm^7^ (Figure 2A). Therefore, we wanted to elucidate whether the allergy‐related differences seen in conventional T cell activation correlated with variations in the unconventional lymphocyte compartment. All the analyzed cell types showed a consistent pattern, characterized by a significantly lower expression of IFN‐γ (Figure 2B) and TNF (Figure S2B) in allergic women. Furthermore, analyzing the longitudinal response of unconventional lymphocytes to SEA in pregnant allergic women, we identified significantly higher production of both IFN‐γ (Figure 2C) and TNF (Figure S2C) across all the studied cell types. Interestingly, this pregnancy‐related increase in the production of both cytokines was absent in the longitudinal samples from non‐allergic women, where the levels were comparable in and out of pregnancy, except for TNF produced by γδ T cells (Figure S2D,E). Overall, these findings underscore the nuanced interplay between conventional and unconventional lymphocytes in the context of SEA exposure and pregnancy. APCs are the first cells to encounter SEA, but very little is known regarding their activation upon superantigen encounter. We here observed that, upon SEA stimulation, the phenotypic marker CD14 was downregulated in all groups, most prominently on non‐pregnant, non‐allergic individuals, as was CD163. CD16 was also downregulated in all groups except for the non‐pregnant non‐allergic (Figure 2D). When analysing T cell interaction markers, we observed that HLADR transcription was downregulated in non‐pregnant non‐allergic individuals, whereas its transcription remained stable in all other groups. Other factors such as CD80 and CD274 (PDL1) were similarly upregulated in all groups. Transcription of type‐1 associated cytokine IL12 showed high variability among individuals in all groups (Figure 2E).

**FIGURE 2:**
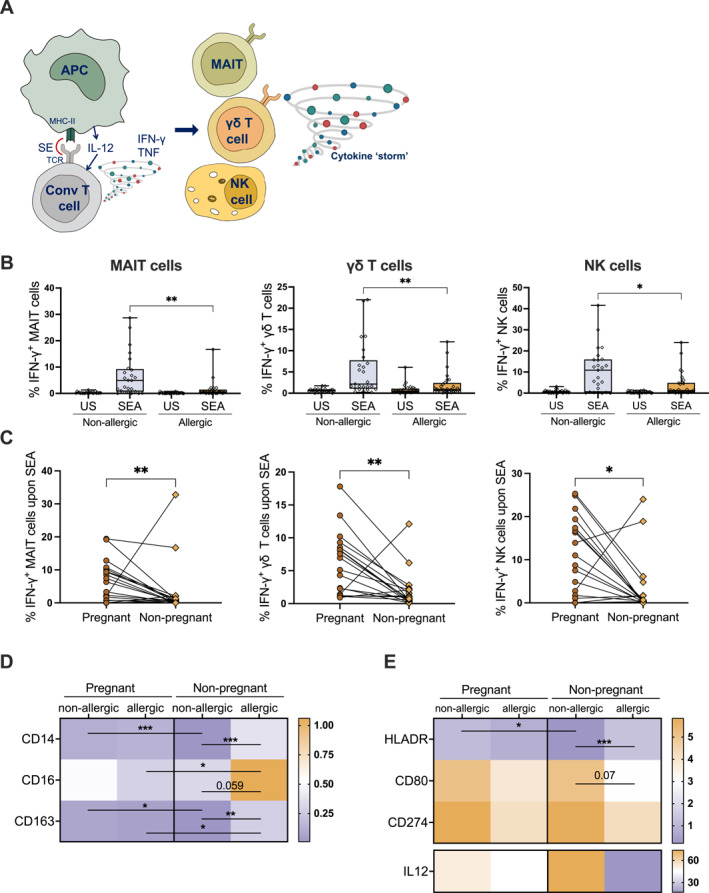
Unconventional lymphocyte and APC responses to SEA. (A) Schematic representation of the contribution of APCs, conventional T‐cells and unconventional lymphocytes in the response to SEA. (B) Proportion of IFN‐γ‐producing MAIT cells, γδ T cells and NK cells. (C) Longitudinal assessment of the IFN‐γ production upon SEA during and out of pregnancy in MAIT, γδ T and NK cells in allergic women. (D) Transcriptional activation of CD14, CD16, CD163, HLADR, CD80, CD274 and IL12 in PBMCs, normalized to a housekeeping gene. (*n* = 5–28).

We are the first to show that allergic women have a suppressed type 1 immune response to SEA, which is restored during pregnancy and is attributed to the strong response by unconventional lymphocytes. An understanding of the trajectory of immune alterations is crucial to optimize the vigilance and therapy strategies in the face of environmental exposures such as SEA, to safeguard the health of both mother and child.

## AUTHOR CONTRIBUTIONS

Study design: Eva Sverremark‐Ekström, Caroline Nilsson, Claudia Arasa; Funding acquisition: Eva Sverremark‐Ekström, Caroline Nilsson; Patient inclusion and sample collection: Caroline Nilsson; Experimental design: Claudia Arasa, Eva Sverremark‐Ekström; Experimental work: Claudia Arasa, Niamh Hyland; Data analysis: Claudia Arasa, Niamh Hyland; Data interpretation: Claudia Arasa, Eva Sverremark‐Ekström. Writing of the manuscript: Claudia Arasa; Critical review of the manuscript: All co‐authors.

## CONFLICT OF INTEREST STATEMENT

ESE has received honoraria for lectures and a grant for another research project from BioGaia AB. CN report grants to institution from Aimmune Therapeutics a Nestlé Company and Lecture fees from: MEDA ALK; Thermofisher and GSK. The other authors have no conflict of interest to declare.

## Supporting information

Supporting Information S1

Figure S1

Figure S2

Figure S3

Table S1

Table S2

Table S3

## Data Availability

The data supporting the findings of this study are available on request from the corresponding author. The data are not publicly available due to privacy or ethical restrictions.
