# Supplementary material for: Pregnancy impacts allergy‐related differences in the response to a type‐1 stimulus, staphylococcal enterotoxin A
Source: Clin Transl Allergy. 2024 Oct 26;14(10):e70007. doi: 10.1002/clt2.70007 (PMC11512600; doi:10.1002/clt2.70007)
Supplement: Supplementary file 1 — Supporting Information S1 [file CLT2-14-e70007-s002.docx]

**Supplementary material**

**Materials and methods**

**Subjects and Ethical Statement**

This study used samples from the mothers in a prospective cohort of 281 children born into the study between 1997 and 2000 in Stockholm, Sweden. The cohort has been described in detail elsewhere^24^. Briefly, families living in Stockholm and expecting a child were invited to participate in the study. Allergic status of the mothers was characterized and only confirmed negative or positive skin prick test results against pollen/furred pets were included. For a short summary of the subjects used in this study see **Table 1**. Samples presented here were taken from mothers at two timepoints, during the third trimester of pregnancy (around week 35) and two years postpartum.

The study was approved by the Human Ethics Committee at Huddinge University Hospital, Stockholm and the patients provided informed verbal consent. No written documentation of the participants’ informed approval was required, which was agreed to by the Human Ethics Committee and was according to the regulations at the time of the initiation of the study.

**Isolation of peripheral blood mononuclear cells (PBMCs)**

Venous blood was diluted in cell culture medium (RPMI-1640 cell culture medium, 20 mM HEPES) and then PBMCs isolated by Ficoll-Hypaque (GE Healthcare Bio-Sciences AB) gradient separation. PBMCs were washed in RPMI-1640 and resuspended in freezing medium (40% RPMI-1640, 50% fetal calf serum (FCS), 10% DMSO), gradually frozen in a freezing container (Mr Frosty, Nalgene Cryo 1°C, Nalgene Co.) and stored in liquid nitrogen until used.

***In vitro* stimulation of PBMCs**

Frozen PBMCs were thawed, washed and resuspended in washing media (RPMI-1640 cell culture medium, 20 mM HEPES), and live cells were counted using trypan blue staining. PBMCs were diluted in cell culture medium (RPMI-1640, 20 mM HEPES, 100 U/mL penicillin, 100 mg/mL (all from HyClone Laboratories, Inc.) and FCS 10% (Gibco by Life Technologies) to a final concentration of 1x10^6^ cells/mL. Cells were seeded into a 48-well flat-bottomed culture plate (Costar® Corning Inc.) and stimulated with the appropriate stimuli; 20 ng/mL purified SEA (Toxin Technology Inc.) or cell culture medium control. Cells were incubated at 37°C, 5% CO_2_atmosphere for 48 hours. Following stimulation, the cells were collected for flow cytometry or Reverse Transcriptase (RT)-qPCR analysis, and cell-free supernatant was collected and frozen at -20°C for later use.

**Flow Cytometry**

Different panels were used for different analysis (see **Table 2**). For the unconventional lymphocyte panel, Brefeldin A (BD Golgi Plug™, BD Biosciences) was added during the last 4 hours of stimulation to prevent protein secretion. After incubation, cells were harvested, transferred to V-shaped staining plates and washed twice in PBS. Cells were stained with the BD Horizon™ Fixable Viability Stain 780 (FVS780) (BD Biosciences) for 15 minutes at room temperature and then washed with PBS. Afterwards, cells were incubated with 10% human serum in FACS wash buffer (PBS, 2 mM EDTA and 0.1% BSA (Roche Diagnostics)) for 10 minutes at 4°C to block Fc-receptors. Cells were then stained for surface markers diluted in FACS wash buffer. After the extracellular staining, cells were washed and fixed/permeabilized using Fix/Perm Buffer (BioLegend) according to manufacturer’s instructions and then blocked with 10% human serum in Perm/Wash Buffer (BioLegend) for 10 minutes at 4°C. Cells were then stained intracellularly (**Table 2A**).

For the Th polarization panel, cells were stained with LIVE/DEAD^TM^ Fixable Aqua Dead Cell Stain kit (Life Technologies) diluted in PBS, and Fc-receptors were blocked with 10% human serum in FACS wash buffer. The cells were subsequently stained extracellularly. After extracellular staining, cells were fixed and permeabilized with True-Nuclear Transcription Factor Buffer Set (BioLegend), according to instructions from the manufacturer. Intracellular blocking was performed using 10% human serum in permeabilization buffer, and then cells were stained intranuclearly with antibodies also diluted in permeabilization buffer (**Table 2B**).

Stained cells from both panels were finally washed in FACS-wash buffer and analysed using the FACSVerse instrument and FACSSuite software (both from BD Biosciences). Unstimulated cells were used as negative controls. Analysis was performed using the FlowJo Software (TreeStar). See the complete gating strategy for the unconventional lymphocyte panel and the Th polarization panel on **Supplementary Figure 1A** and **1B** respectively.

**Reverse Transcriptase (RT) qPCR**

Total RNA from PBMC stimulated for 48h were harvested using the Quick-RNA^TM^ MiniPrep kit (Zymo Research, BioLegend) following manufacturer’s instructions. From the extracted RNA, cDNA transcripts were obtained using SuperScript VILO cDNA Synthesis kit (Thermo Fischer Scientific). RT-qPCR was performed on a LightCycler® 480 II Real-Time PCR instrument (Roche Diagnostics) using gene-specific primers (**Table 3**) and KAPA SYBR FAST qPCR Master Mix (KAPA Biosystems Inc.). Target gene expression was normalized to the expression of the housekeeping gene 18S.

**ELISA**

Cell-free supernatants from stimulated PBMCs were thawed and cytokine secretion was measured by ELISA. Levels of IFN-γ, IL-5, IL-10, IL-13 or IL-17A (all from MabTech AB) were determined by sandwich ELISA according to manufacturer’s instructions. The optical density at 405nm was determined using a micro-plate reader (Molecular Devices Corp). Results were analyzed using SoftMax Pro 5.2 rev C (Molecular Devices Corp).

**Statistical analysis**

The statistical analysis and presentation were performed in GraphPad Prism 8 software (GraphPad Software La Jolla, CA, USA). Nonparametric statistical analyses were performed on all datasets. The Mann-Whitney U test was used to compare the response by relevant groups; and the Wilcoxon matched-pairs signed rank test was used on longitudinal comparisons of 2 parameters within the same individual. All graphs display median with interquartile range, with boxes showing the median as a central line and covering the 25^th^ to 75^th^ percentile, and symbols indicating individual values. Differences were considered significant when P-values < 0.05. The significance levels used were **p*< 0.05, ***p*< 0.01, ****p*< 0.001 and **** *p* < 0.0001. Number of donors (n) in each experiment is specified in the figure legends.

**Figure legends**

**Supplementary Figure 1. T cell responses to SEA.** (A) Secretion of type 1 cytokines by PBMCs from pregnant women. (B) Secretion of type 2 cytokines by PBMCs from non-pregnant women. (C) Ratio of CD4+ T-cells expressing RORγt normalized to the unstimulated (left); IL-17A secretion (right). (D) Ratio of CD4+ T-cells expressing FoxP3 normalized to the unstimulated (left); IL-10 secretion (right). (n=5-28)

**Supplementary Figure 2. Unconventional lymphocyte responses to SEA.** (A) Proportion of TNF-producing conventional T cells outside of pregnancy, and longitudinal assessment of the TNF production upon SEA during (**○**) and out of pregnancy (⬦) in non-allergic and allergic women. (B) Proportion of TNF-producing MAIT, γδ T and NK cells outside of pregnancy. Longitudinal assessment of the SEA response in terms of TNF production in allergic women (C); and IFN-γ (D) and TNF (E) in non-allergic women within MAIT, γδ T and NK cells during (**○**) and out of pregnancy (⬦). (n=13-28)

**Supplementary Figure 3. Gating strategy.** Lymphocytes were gated based on their forward- and side scatter properties. (A) Single cells were selected within the Live cells. These were then gated based on specific cell markers: NK cells were CD56+ CD3-; CD3+ T cells were further gated into Panγδ TCR+ cells (γδ T cells), and Panγδ TCR- cells were separated into CD161+ Vα7.2+ cells (MAIT cells) whereas the rest were considered conventional T cells. Intracellular expression of IFN-γ or TNF was assessed within each population. (B) T helper cells were gated from single live cells, according to their CD4+ expression. They were then further gated according to their expression of Tbet, GATA3, RORγt or FoxP3.

**Additional references**

Bachert, C., Humbert, M., Hanania, N. A., Zhang, N., Holgate, S., Buhl, R., & Bröker, B. M. (n.d.). *Staphylococcus aureus and its IgE-inducing enterotoxins in asthma: current knowledge STATE OF THE ART ASTHMA*. https://doi.org/10.1183/13993003.01592-2019

Baghlaf, H., Spence, A. R., Czuzoj-Shulman, N., & Abenhaim, H. A. (2019). Pregnancy outcomes among women with asthma. *Journal of Maternal-Fetal and Neonatal Medicine*, *32*(8), 1325–1331. https://doi.org/10.1080/14767058.2017.1404982

Björkstén, B., Sepp, E., Julge, K., Voor, T., & Mikelsaar, M. (2001). Allergy development and the intestinal microflora during the first year of life. *Journal in Allergy and Clinical Immunology*, *108*(4), 516–520. Retrieved from https://www.jacionline.org/action/showPdf?pii=S0091-6749%2801%2996140-8

Borzychowski, A. M., Croy, B. A., Chan, W. L., Redman, C. W. G., & Sargent, I. L. (n.d.). *Changes in systemic type 1 and type 2 immunity in normal pregnancy and pre-eclampsia may be mediated by natural killer cells*. https://doi.org/10.1002/eji.200425929

Colucci, F. (2019). The immunological code of pregnancy. *Science*, *365*(6456), 862–863. https://doi.org/10.1126/science.aaw1300

Doster, R. S., Kirk, L. A., Tetz, L. M., Rogers, L. M., Aronoff, D. M., & Gaddy, J. A. (2017). *Staphylococcus aureus Infection of Human Gestational Membranes Induces Bacterial Biofilm Formation and Host Production of Cytokines*. 653. https://doi.org/10.1093/infdis/jiw300

Holgate, S. T. (2008). Pathogenesis of Asthma. *Clinical & Experimental Allergy*, *38*(6), 872–897. https://doi.org/10.1111/J.1365-2222.2008.02971.X

Holtfreter, S., Roschack, K., Eichler, P., Eske, K., Holtfreter, B., Kohler, C., … Bröker, B. M. (2005). Staphylococcus aureus Carriers Neutralize Superantigens by Antibodies Specific for Their Colonizing Strain: A Potential Explanation for Their Improved Prognosis in Severe Sepsis. In *The Journal of Infectious Diseases* (Vol. 209). Retrieved from https://academic.oup.com/jid/article/193/9/1275/1013493

Jamieson, D. J., Theiler, R. N., & Rasmussen, S. A. (2006). Emerging infections and pregnancy. *Emerging Infectious Diseases*. https://doi.org/10.3201/eid1211.060152

Kourtis, A. P., Read, J. S., & Jamieson, D. J. (2014). Pregnancy and Infection. *New England Journal of Medicine*, *370*(23), 2211–2218. https://doi.org/10.1056/NEJMRA1213566

Mor, G., Aldo, P., & Alvero, A. B. (2017). The unique immunological and microbial aspects of pregnancy. *Nature Reviews Immunology*, *17*(8), 469–482. https://doi.org/10.1038/nri.2017.64

Mor, G., & Cardenas, I. (n.d.). *The Immune System in Pregnancy: A Unique Complexity*. https://doi.org/10.1111/j.1600-0897.2010.00836.x

Mor, G., & Cardenas, I. (2010). The Immune System in Pregnancy: A Unique Complexity. *American Journal of Reproductive Immunology*, *63*, 425–433. https://doi.org/10.1111/j.1600-0897.2010.00836.x

Morelli, M. K., Veve, M. P., & Shorman, M. A. (2020). Maternal bacteremia caused by Staphylococcus Aureus with a focus on infective endocarditis. *Open Forum Infectious Diseases*, *7*(8). https://doi.org/10.1093/ofid/ofaa239

Neeland, M. R., Andorf, S., Dang, T. D., McWilliam, V. L., Perrett, K. P., Koplin, J. J., & Saffery, R. (2021). Altered immune cell profiles and impaired CD4 T-cell activation in single and multi-food allergic adolescents. *Clinical and Experimental Allergy*, *51*(5), 674–684. https://doi.org/10.1111/cea.13857

Nordengrün, M., Michalik, S., Völker, U., Bröker, B. M., & Gómez-Gascón, L. (2018). The quest for bacterial allergens. *International Journal of Medical Microbiology*, *308*(6), 738–750. https://doi.org/10.1016/J.IJMM.2018.04.003

Nowrouzian, F. L., Lina, G., Hodille, E., Lindberg, E., Hesselmar, B., Saalman, R., … Wold, A. E. (2017). Superantigens and adhesins of infant gut commensal Staphylococcus aureus strains and association with subsequent development of atopic eczema. *British Journal of Dermatology*, *176*(2), 439–445. https://doi.org/10.1111/bjd.15138

Paul, W. E., & Zhu, J. (2010). *How are TH2-type immune responses initiated and amplified?* https://doi.org/10.1038/nri2735

Petursdottir, D. H., Nordlander, S., Qazi, K. R., Carvalho-Queiroz, C., Osman, O. A., Hell, E., … Sverremark-Ekström, E. (2017). Early-life human microbiota associated with childhood allergy promotes the T helper 17 axis in mice. *Frontiers in Immunology*, *8*(DEC), 1–14. https://doi.org/10.3389/fimmu.2017.01699

Pfaller, B., Bendien, S., Ditisheim, A., & Eiwegger, T. (2022). Management of allergic diseases in pregnancy. *Allergy: European Journal of Allergy and Clinical Immunology*, *77*(3), 798–811. https://doi.org/10.1111/all.15063

Romanowska-Próchnicka, K., Felis-Giemza, A., Olesińska, M., Wojdasiewicz, P., Paradowska-Gorycka, A., & Szukiewicz, D. (2021). The role of tnf-α and anti-tnf-α agents during preconception, pregnancy, and breastfeeding. *International Journal of Molecular Sciences*, Vol. 22, pp. 1–22. https://doi.org/10.3390/ijms22062922

Saito, S. (2000). Cytokine network at the feto-maternal interface. *Journal of Reproductive Immunology*, *47*(2), 87–103. https://doi.org/10.1016/S0165-0378(00)00060-7

Sintobin, I., Keil, T., Lau, S., Grabenhenrich, L., Holtappels, G., Reich, A., … Bachert, C. (2015). Is immunoglobulin E to Staphylococcus aureus enterotoxins associated with asthma at 20 years? *Pediatric Allergy and Immunology*, *26*(5), 461–465. https://doi.org/10.1111/pai.12396

Szekeres-Bartho, J., & Wegmann, T. G. (1996). A progesterone-dependent immunomodulatory protein alters the Th1/Th2 balance. *Journal of Reproductive Immunology*, *31*(1–2), 81–95. https://doi.org/10.1016/0165-0378(96)00964-3

Tanaka, A., Suzuki, S., Ohta, S., Manabe, R., Furukawa, H., Kuwahara, N., … Sagara, H. (2015). Association between specific IgE to Staphylococcus aureus enterotoxins A and B and asthma control. *Annals of Allergy, Asthma and Immunology*, *115*(3), 191-197.e2. https://doi.org/10.1016/j.anai.2015.06.017

Tulic, M. K., Hodder, M., Forsberg, A., McCarthy, S., Richman, T., DVaz, N., … Prescott, S. L. (2011). Differences in innate immune function between allergic and nonallergic children: New insights into immune ontogeny. *Journal of Allergy and Clinical Immunology*, *127*(2), 470-478.e1. https://doi.org/10.1016/j.jaci.2010.09.020

Watanabe, M., Iwatani, Y., Kaneda, T., Hidaka, Y., Mitsuda, N., Morimoto, Y., & Amino, N. (1997). Changes in T, B, and NK lymphocyte subsets during and after normal pregnancy. *American Journal of Reproductive Immunology*, *37*(5), 368–377. https://doi.org/10.1111/j.1600-0897.1997.tb00246.x

Weetman, A. P. (1999). Immunology of pregnancy. *Thyroid*, *9*(7), 643–646. https://doi.org/10.5005/jp/books/12974_10

Wegmann, T. G. (1984). Foetal protection against abortion: Is it immunosuppression or immunostimulation? *Annales de l’Institut Pasteur - Immunology*, *135*(3), 309–312. https://doi.org/10.1016/S0769-2625(84)81196-4

Wegmann, Thomas G., Lin, H., Guilbert, L., & Mosmann, T. R. (1993). Bidirectional cytokine interactions in the maternal-fetal relationship: is successful pregnancy a TH2 phenomenon? *Immunology Today*, *14*(7), 353–356. https://doi.org/10.1016/0167-5699(93)90235-D

Zeng, W. ping, McFarland, M. M., Zhou, B., Holtfreter, S., Flesher, S., Cheung, A., & Mallick, A. (2017). Staphylococcal enterotoxin A–activated regulatory T cells promote allergen-specific TH2 response to intratracheal allergen inoculation. *Journal of Allergy and Clinical Immunology*, *139*(2), 508-518.e4. https://doi.org/10.1016/j.jaci.2016.04.033
