# Supplementary figures and images for: Pregnancy impacts allergy‐related differences in the response to a type‐1 stimulus, staphylococcal enterotoxin A
Source: Clin Transl Allergy. 2024 Oct 26;14(10):e70007. doi: 10.1002/clt2.70007 (PMC11512600; doi:10.1002/clt2.70007)

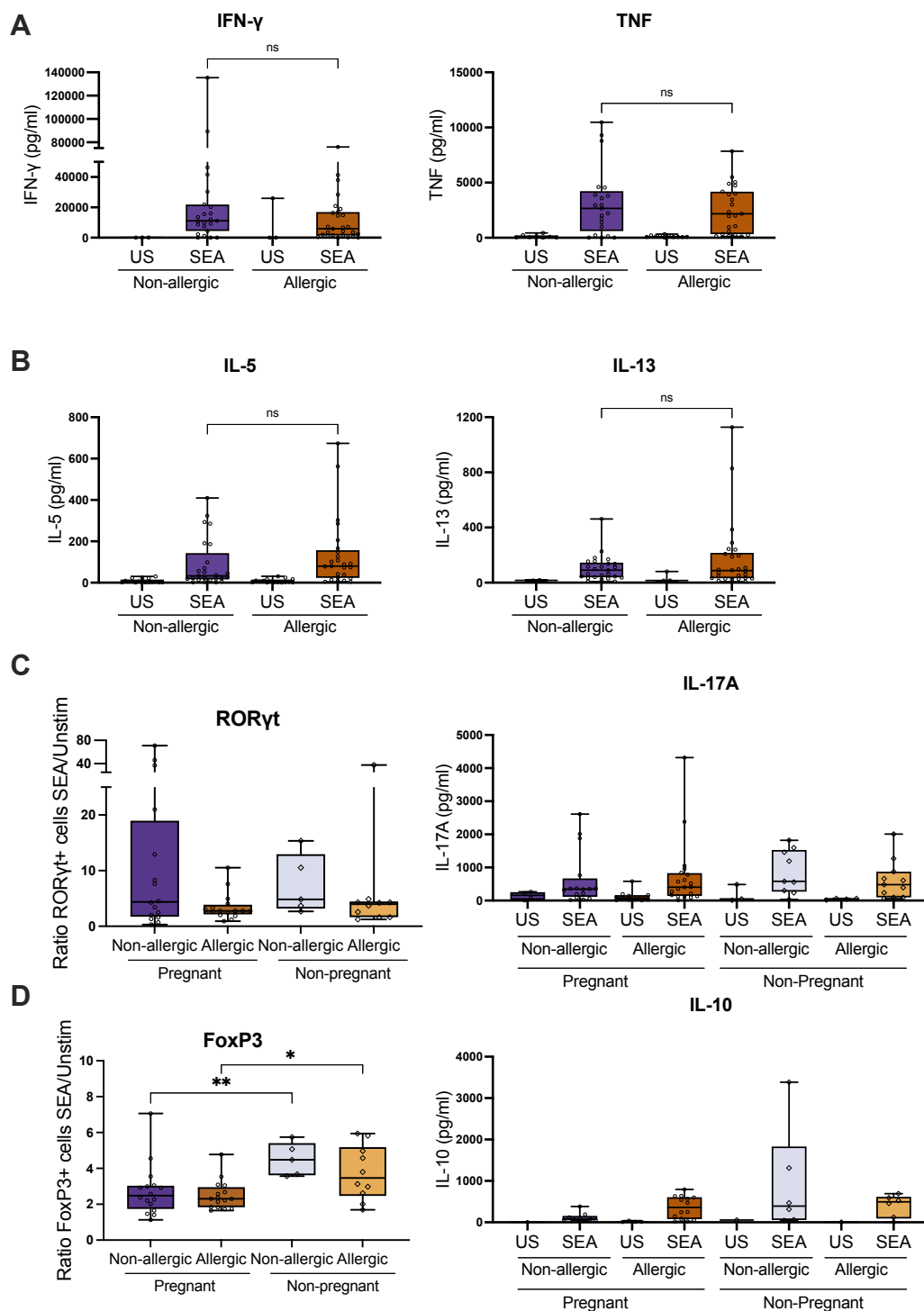

Supplement: Supplementary file 2 — Figure S1 [file CLT2-14-e70007-s001.pdf]

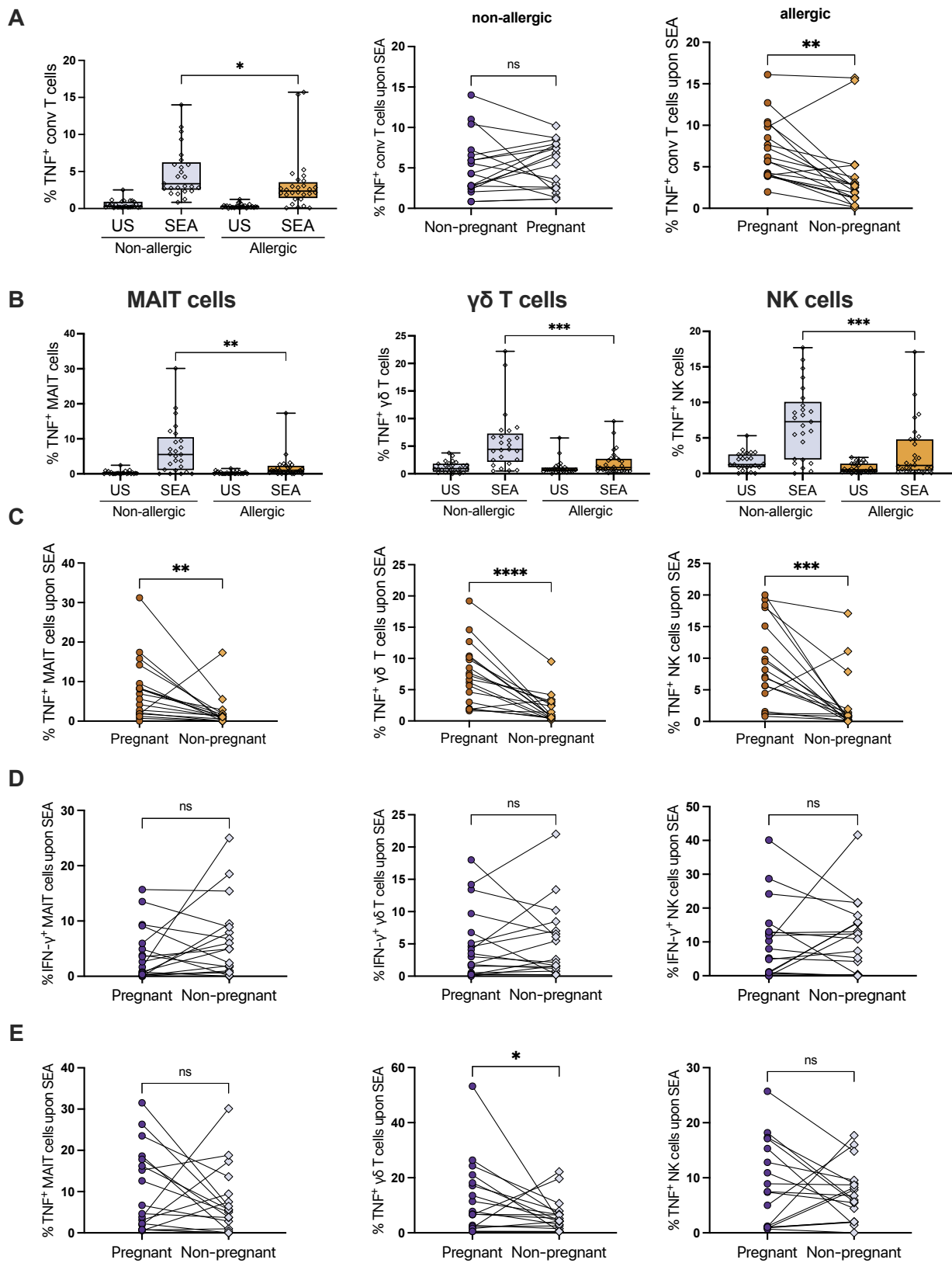

Supplement: Supplementary file 3 — Figure S2 [file CLT2-14-e70007-s005.pdf]

**A**

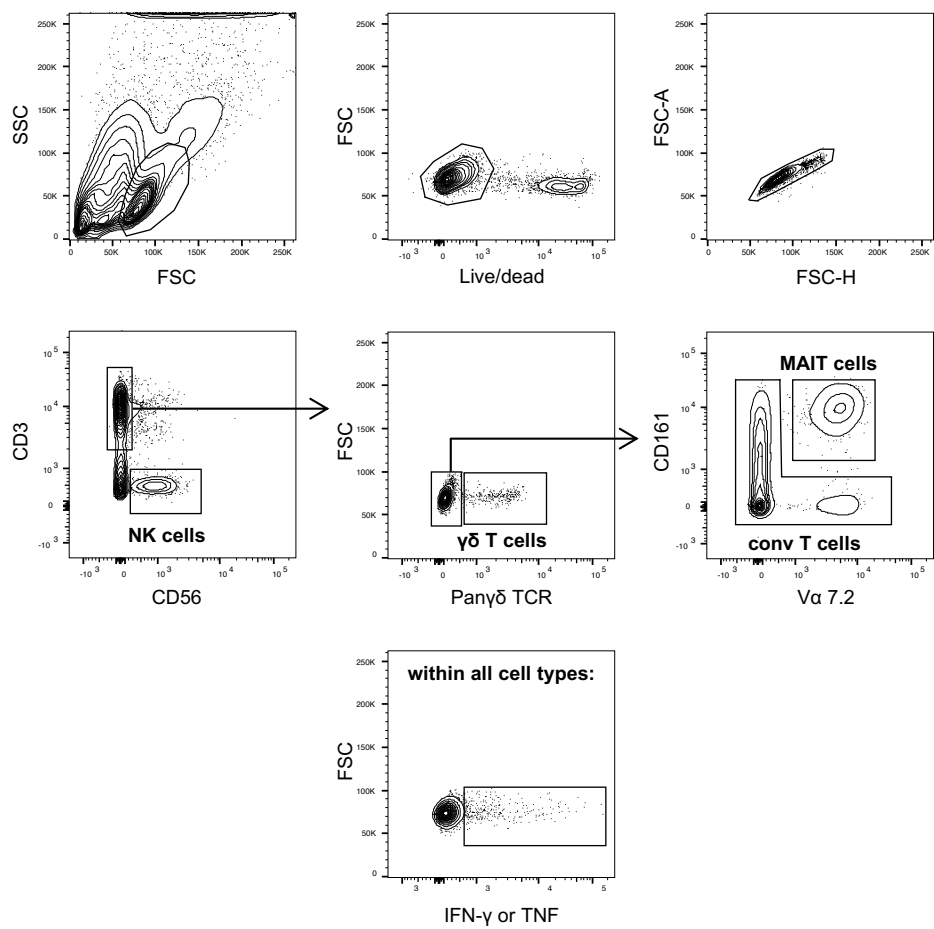

**B**

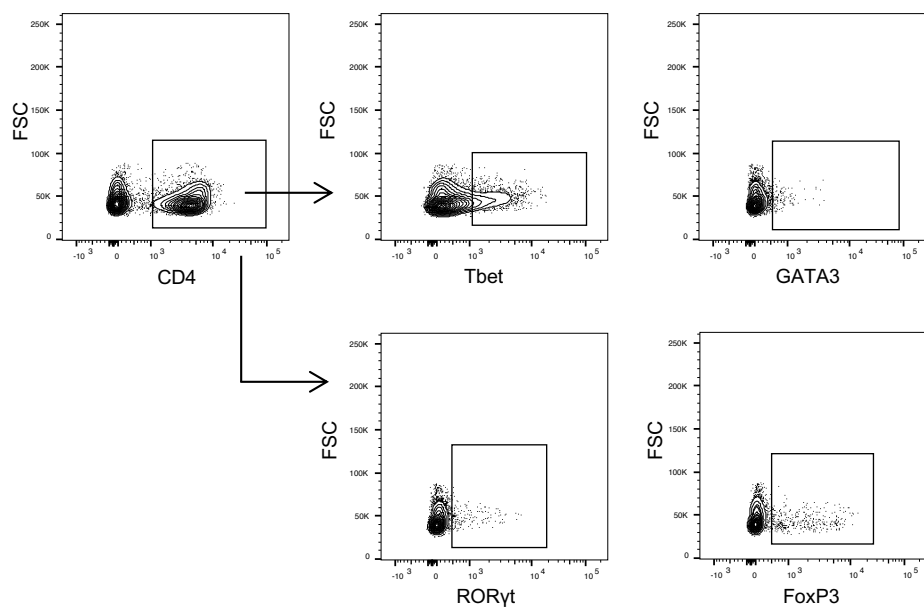

Supplement: Supplementary file 4 — Figure S3 [file CLT2-14-e70007-s004.pdf]
