# Supplementary material for: Pregnancy impacts allergy‐related differences in the response to a type‐1 stimulus, staphylococcal enterotoxin A
Source: Clin Transl Allergy. 2024 Oct 26;14(10):e70007. doi: 10.1002/clt2.70007 (PMC11512600; doi:10.1002/clt2.70007)
Supplement: Supplementary file 5 — Table S1 [file CLT2-14-e70007-s007.pdf]

Table 1. Demographic data of allergic and non-allergic women.

	**Allergic** (n=35)	**Non-allergic** (n=30)	**P value** (Mann-Whitney rank comparison)
Maternal age at delivery [years; mean (range)]	33 (21-44)	31(23-40)	0.08
Smoking [number (percentage)]	1 (2.86%)	3 (10%)	0.33
Gestational week [weeks; mean (range)]	40 (36-42)	40 (38-43)	0.23
Sex of the child	Girl 23/35<br>Boy 12/35	Girl 14/30<br>Boy 16/30	0.14
**Allergies** [number (percentage)]			
Pollen allergy	26 (74.29%)	0 (0%)	
Animal fur	22 (62.86)	0 (0%)	
Food allergy	14 (40%)	0 (0%)	
Mold/mites	6 (17.14%)	0 (0%)	
Latex	1 (2.86%)	0 (0%)	
**Allergic symptoms** [number (percentage)]			
Asthma	14 (40%)	0 (0%)	
Rhinitis	30 (85.71%)	0 (0%)	
Conjunctivitis	26 (74.29%)	0 (0%)	
Eczema	10 (28.57%)	0 (0%)	
Urticaria	3 (8.57%)	0 (0%)	
Mouth/throat itching	20 (57.14%)	0 (0%)	
