# Supplementary material for: Pregnancy impacts allergy‐related differences in the response to a type‐1 stimulus, staphylococcal enterotoxin A
Source: Clin Transl Allergy. 2024 Oct 26;14(10):e70007. doi: 10.1002/clt2.70007 (PMC11512600; doi:10.1002/clt2.70007)
Supplement: Supplementary file 6 — Table S2 [file CLT2-14-e70007-s006.pdf]

Table 2. Overview of the flow cytometry panels

Panel	Marker	Fluorochrome	Clone	Staining	Company
**A.** **Unconventional lymphocyte**	CD3	BV521	UCHT1	surface	BioLegend
	CD56	APC	B159	surface	BD Biosciences
	CD161	PE-Cy7	HP-3G10	surface	BioLegend
	Vα7.2	PE	3C10	surface	BioLegend
	pan-γδ TCR	FITC	CE-IVD	surface	Beckman Coulter
	IFN-γ	PerCP Cy5.5	B27	intracellular	BD Biosciences
	TNF	BV421	MAb 11	intracellular	BD Biosciences
**B.** **Th polarization**	CD4	PerCP Cy5.5	OKT4	surface	BioLegend
	Tbet	BV421	16E10A23	intranuclear	BioLegend
	GATA3	Alexa Fluor 647	150D	intranuclear	BioLegend
	RORγt	PE	Q21-559	intranuclear	BD Biosciences
	FoxP3	Alexa Fluor 488	4B10	intranuclear	BioLegend
