# Supplementary material for: Pregnancy impacts allergy‐related differences in the response to a type‐1 stimulus, staphylococcal enterotoxin A
Source: Clin Transl Allergy. 2024 Oct 26;14(10):e70007. doi: 10.1002/clt2.70007 (PMC11512600; doi:10.1002/clt2.70007)
Supplement: Supplementary file 7 — Table S3 [file CLT2-14-e70007-s003.pdf]

Table 3. Primer sequences used for RT-qPCR

Gene	Primer sequence	Company
18S	F – CCATCCAATCGGTAGTAGCG<br>R – GTAACCCGTTGAACCCATT	Eurofins Genomics
CD14	F – GATTACATAAACTGTCAGAGGC<br>R – TCCATGGTCGATAAGTCTTC	Merck (Sigma Aldrich)
CD16a	F – CGGATATCTTTGGTGACTTG<br>R – AACTAGAAGTAGCAGAGCAG	Merck (Sigma Aldrich)
CD80	F – AGGAGGAATGAGAGATTGAG<br>R – GACCTTCAGATCTTTTCAGC	Merck (Sigma Aldrich)
CD163	F – ATGAGTCCCATCTTTCACTC<br>R – CTATGTCCCAGTGAGAGTTAC	Merck (Sigma Aldrich)
CD274	F – ATGCCCCATACAACAAAATC<br>R – GACATGTCAGTTCATGTTCAG	Merck (Sigma Aldrich)
FOXP3	F – ACATTCCCAGAGTTCCTC<br>R – AGATCTCATTGAGTGTCCG	Merck (Sigma Aldrich)
GATA3	F – AAAATGAACGGACAGAACC<br>R – GGGGTCTGTTAATATTGTGAAG	Merck (Sigma Aldrich)
HLADR	F – CATTATTGGGACCATCTTC<br>R – TAAGAAACACCATCACCTCC	Merck (Sigma Aldrich)
IL12	F – CCATTGAGGTCATGGTGG<br>R – ACGCAGAATGTCAGGGAGAA	Eurofins Genomics
RORC	F – TTTTGAAGGCAAATACGGTG<br>R – AGTGGGAGAAGTCAAAGATG	Merck (Sigma Aldrich)
TBX21	F – CTTTCCAAGAAACCCAGTTC<br>R – GTCAACAGATGTGTACATGG	Merck (Sigma Aldrich)
